# Diagnosing Sport-Related Flow Limitations in the Iliac Arteries Using Near-Infrared Spectroscopy

**DOI:** 10.3390/jcm11247462

**Published:** 2022-12-16

**Authors:** Martijn van Hooff, Jem Arnold, Eduard Meijer, Paul Schreuder, Marta Regis, Lin Xu, Marc Scheltinga, Hans Savelberg, Goof Schep

**Affiliations:** 1 Department of Sports and Exercise, Máxima Medical Centre, De Run 4600, 5500 MB Veldhoven, The Netherlands; 2 Department of Nutrition and Movement Science, NUTRIM School for Nutrition and Translational Research in Metabolism, Faculty of Health, Medicine and Life Sciences, Maastricht University, 6200 MD Maastricht, The Netherlands; 3 Environmental Physiology Laboratory, School of Kinesiology, The University of British Columbia, Vancouver, BC V6T 1Z1, Canada; 4 Department of Clinical Physics, Máxima Medical Centre, De Run 4600, 5500 MB Veldhoven, The Netherlands; 5 Department of Mathematics and Computer Science, Eindhoven University of Technology, Den Dolech 2, 5612 AZ Eindhoven, The Netherlands; 6 School of Information Science and Technology, Shanghai Tech University, 393 Middle Huaxia Road, Shanghai 201210, China; 7 Department of Vascular Surgery, Máxima Medical Centre, De Run 4600, 5500 MB Veldhoven, The Netherlands

**Keywords:** muscle oxygen saturation, endofibrosis, maximal exercise testing, near-infrared spectroscopy

## Abstract

Background: A flow limitation in the iliac arteries (FLIA) in endurance athletes is notoriously difficult to diagnose with the currently available diagnostic tools. At present, a commonly used diagnostic measure is a decrease in ankle brachial index with flex hips (ABI_Flexed_) following a maximal effort exercise test. Near-infrared spectroscopy (NIRS) is a non-invasive technique that measures skeletal muscle oxygenation as reflected by the balance of O_2_ delivery from microvascular blood flow and O_2_ uptake by metabolic activity. Therefore, NIRS potentially serves as a novel technique for diagnosing FLIA. The purpose of this study is to compare the diagnostic accuracy of NIRS-derived absolute, amplitude, and kinetic variables in legs during and after a maximal exercise test with ABI_Flexed_. Methods: ABI_Flexed_ and NIRS were studied in 33 healthy subjects and 201 patients with FLIA diagnosed with echo-Doppler. Results: After maximal exercise, NIRS kinetic variables, such as the half value time and mean response time, resulted in a range of 0.921 to 0.939 AUC for the diagnosis of FLIA when combined with ABI_Flexed_. Conversely, ABI_Flexed_ measurements alone conferred significantly worse test characteristics (AUC 0.717, *p* < 0.001). Conclusions: NIRS may serve as a diagnostic adjunct in patients with possible FLIA.

## 1. Introduction

Exercise-induced flow limitations in the iliac artery (FLIA) is a fairly uncommon condition in which arterial blood supply to leg muscles of healthy athletes is impaired during strenuous exercise [1]. FLIA caused simply by arterial kinking is often subtle and more difficult to diagnose than FLIA due to substantial intravascular abnormalities. For instance, an early stage of functional isolated arterial kinking has a low diagnostic sensitivity of 0.73 if comparison to an asymptomatic other leg is possible and only 0.43 if the complaints are bilateral [2,3]. By contrast, more advanced stages of the disease with predominantly intravascular abnormalities are determined with a 0.90 sensitivity [4]. If FLIA caused by arterial kinking remains undetected, intravascular lesions may progressively develop and may occasionally even lead to an occluded artery [5,6].

If symptomatic FLIA is detected at an early stage, conservative treatment is attempted by adapting the sports activities and/or body posture. The early detection of FLIA may allow for less invasive surgery, such as a vascular release procedure [7,8,9,10]. In advanced stages, however, a vascular reconstruction encompassing shortening and/or a venous patch is often indicated. Therefore, a better diagnostic and monitoring tool with optimal diagnostic characteristics in both early and advanced stages of FLIA is of paramount clinical importance.

Near-infrared spectroscopy (NIRS) uses specific wavelengths of light to optically estimate the relative oxygenation and deoxygenation of hemoglobin and myoglobin content within the volume of tissue illuminated by the sensor [11]. Measurements can be performed non-invasively during and after exercise [12,13]. This technique has been used for the diagnosis of peripheral vascular disease [14,15,16,17]. A pilot and case study in FLIA showed promising results [5,18]. A high to near-perfect test–retest reliability of NIRS-derived reoxygenation kinetics after maximal exercise was recently reported [19].

The goal of this study is to investigate the diagnostic sensitivity and specificity of NIRS in athletes with FLIA. As such, in this analysis of clinical patient data, we hypothesized that including NIRS in the diagnostic pathway would improve diagnostic accuracy.

## 2. Materials and Methods

### 2.1. Patient and Healthy Athletes

Recruitment occurred from a population of athletes presenting exercise-induced leg pain between 2013 and 2021 at Máxima Medical Centre in Veldhoven (The Netherlands), a center of expertise to which a substantial number of (inter)national patients are referred. They were included if aged ≥18 years and if they had been active in endurance sports for more than three hours a week for at least three years. Inclusion criteria for symptoms of FLIA were the onset of pain, cramp, and/or powerlessness with high-intensity exercise, which disappeared after a few minutes of rest. They were required to have the diagnosis of FLIA demonstrated by a standardized Doppler ultrasound examination, as outlined in detail previously [2,3,7,20]. Most patients from our Kleinloog et al. study on the diagnostic use of pedal power measurements in patients with unilateral FLIA were included in this study as well [21]. Patients were excluded if they had an earlier vascular reconstruction in the iliac region, if they suffered from heart failure (New York Heart Association stage ≥ I), obesity, microvascular abnormalities (e.g., diabetes), atherosclerosis, or orthopedic/neurological conditions potentially limiting exercise capacity. The exclusion criteria were chosen for safety reasons and for potential bias introduced by unexpected responses during the NIRS measurements.

Healthy athletes served as controls and were contacted via cycling clubs, conferences, and through sports science colleagues. They were interviewed to exclude individuals with complaints suggestive of FLIA and additionally underwent a similar standardized Doppler ultrasound examination, excluding signs of asymptomatic FLIA [2,3,20]. High levels of adipose tissue thickness (ATT) are known to influence the accuracy of NIRS measurements of underlying muscular tissue [12,22,23]. The ATT was calculated as half the skinfold thickness measured by a skinfold caliper (Harpenden, Baty International, West Sussex, UK). A <7.5 mm ATT inclusion threshold at the site of NIRS measurement was chosen. The study protocol was approved by the local Research Ethics Committee of Máxima Medical Centre, Veldhoven, The Netherlands. The study was registered in the Dutch trial register (https://www.trialregister.nl identifier: Trial NL8557; accessed on 24 April 2020). The study was conducted according to the Helsinki Declaration of 1964 [24]. All participants gave verbal and written informed consent.

### 2.2. Doppler Ultrasound Examination

Visualization of possible kinking and/or intravascular lesions in the common and external iliac, as well as femoral arteries, was performed in supine position with hips extended and flexed using transversal and axial scans (Terason T3000, Burlington, MA, USA) [2,3,20]. Peak systolic velocity (PSV) was examined in supine position two centimeters caudal to the aortic/iliac bifurcation and along the external iliac artery (and in the common iliac artery if suggestive of an abnormality) with both extended hip (PSV_Extended_) and flexed hip (PSV_Flexed_). Moreover, PSV measurements were performed in the external iliac artery with flexed hips in combination with isometric psoas contraction in supine position (PSV_Psoas_). Normal values for PSV_Extended_ were <1.48 m∙s^−1^ and <1.70 m∙s^−1^ for PSV_Flexed_ and PSV_Psoas_ [2,3,20].

### 2.3. Exercise Testing

A provocative maximal exercise test was performed in competitive posture on an electromagnetically braked cycle-ergometer (Excalibur Sport, Lode, Groningen, The Netherlands). A constant self-selected pedaling frequency between 80 and 100 revolutions per minute was maintained. Prior to the exercise phase, a four-minute warm-up at 10% of their approximated workload (based on sex, bodyweight, and physical fitness) was performed. This was followed by an incremental ramp exercise test at an individualized ramp rate that allowed the subject to reach maximal exercise tolerance (‘exhaustion’) within 8–12 min [25,26]. Maximal task tolerance was determined as either a drop in cadence of >10 rpm for >5 s, despite strong verbal encouragement, or otherwise voluntary cessation. The last registered workload was defined as the peak workload ($W_{\mathrm{Peak}}$). All subjects were advised to avoid excessive exercise 48 h prior to this test.

Once task intolerance was attained, the subject was instructed to quickly place both feet on a resting platform that was positioned over the bike while maintaining the competitive posture. Subsequently, blood pressure was measured during this recovery period at the ankles (Duo, Datascope Corp., Montvale, NJ, USA) and the arm (Critikon 1846-SX, Soma Technology, Highland park Dr., Bloomfield, CO, USA) in order to measure the adapted ankle brachial index (ABI_Flexed_). The hydrostatic pressure, generated by the column of blood between ankle and arm needs to be corrected by measuring the height difference between the arm and ankle [3,27]. The following equation is used:

(1) $${\mathrm{ABI}}_{\mathrm{Flexed}}=\frac{\left(\mathrm{SAP}-\left(\Delta_{\mathrm{AB}}\times 0.78\right)\right)}{\mathrm{SBP}}\;$$

where *SAP* is the systolic ankle pressure in mmHg and $\Delta_{AB}$ is the vertical difference in height between the ankle and arm in cm (1 cm = 0.78 mmHg). The SBP is the systolic brachial pressure in mmHg. Normal ABI_Flexed_ values for competitive athletes in this posture are ≥0.54, or a pressure difference between legs ≤23 mmHg [2,3].

### 2.4. Near-Infrared Spectroscopy

Near-infrared spectroscopy is a non-invasive optical technology that relies on the relative translucency of biological tissue to light in the near-infrared spectrum (700–1300 nm). By using distinct wavelengths with known absorption spectra, NIRS is able to detect relative concentration changes in the oxygenation status of hemoglobin and myoglobin over time [13]. It is known that near-infrared light is absorbed by hemoglobin and myoglobin. Although their absorption cannot be distinguished, both will be referred to as hemoglobin for convenience [11]. From the different absorption properties of oxygenated hemoglobin (O_2_Hb) and deoxygenated hemoglobin (HHb) at different light wavelengths, the relative concentration changes can be computed in the illuminated tissue.

During this study, we used a spatially resolved spectroscopy (SRS) device (Portamon, Artinis, Elst, The Netherlands) with a single detector photo diode and three pairs of light-emitting diodes (LED) at spacings of 30, 35, and 40 mm that emit light at two wavelengths (760 and 850 nm). O_2_Hb and HHb are each resolved at all three inter-optode channel pairings, and relative tissue saturation index (TSI) can be calculated as (TSI = [O_2_Hb]/([O_2_Hb] + [HHb]) × 100). The absolute TSI value analyzed in the present study is calculated from the spatial difference across all three channels, according to photon diffusion theory of SRS-NIRS [28]. By calculating TSI, a reflectance of the dynamic balance between delivery and utilization in the muscle tissue can be examined. Moreover, the differential hemoglobin (dHb) level reflects the net blood oxygenation changes and is calculated as [dHb] = [O_2_Hb] − [HHb] [29]. A sampling frequency of 10 Hz and a differential path length of four was used to correct for effective scattering distance of photons in tissue, as recommended by the manufacturer. Preceding fixation, the NIRS devices were wrapped in cling foil to prevent moisture entering the device. The NIRS devices were positioned on each leg and fixed on the distal belly of the vastus lateralis muscle, 15 cm superior to the proximal patellar rim. A black fabric cover in combination with elastic Velcro was placed over the device preventing gross movements while the influence of ambient light was minimized. This was performed consistently by the same investigator.

### 2.5. Data Analysis

Data were analyzed in a custom-made MatLab program (MatLab R2021a (9.10.0.1602886), Mathworks, Nattick, MA, USA). A Hampel filter was used to detect outliers in the NIRS signal (e.g., from unwanted body movements). These outliers were replaced with the median value if exceeding three standard deviations from the value itself and three neighboring points to each side [30]. Signals were then filtered using a 5th order Butterworth low-pass filter with a 1 Hz cut-off frequency. Zero-lag filtering was used to prevent phase shifting. The end of the cyclic pattern in the signal was considered as the start-point of the recovery phase [5,18,19].

#### 2.5.1. Absolute Values

**Figure 1:**
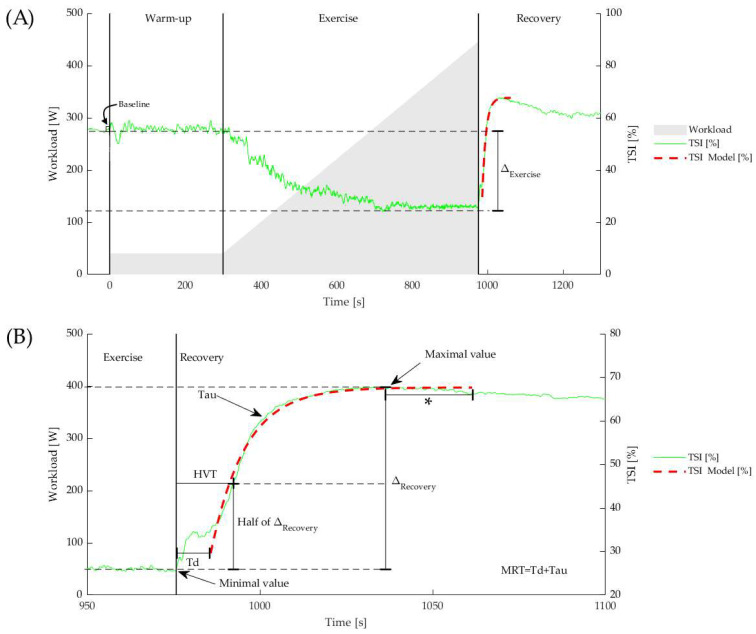
(**A**) Example of TSI changes during and after a maximal exercise test. The vertical lines represent start of warm-up, exercise, and recovery phase. The dashed red curve represents the best fit of the mono-exponential model. (**B**) Td is the time between the start of recovery phase and onset of the mono-exponential model. The MRT is the sum of the Td and the time constant Tau. The HVT was calculated as the time to reach half the $\Delta_{Recovery}$ in the recovery period. * The fitting procedure included a 30 s window following the peak value.

The baseline TSI is calculated as the 10 s average prior to the beginning of the exercise phase. Throughout the exercise phase, TSI, O_2_Hb, and dHb gradually decrease to a minimal value or plateau near maximal exercise. During post-exercise recovery, they all increase to maximal values during the post-exercise hyperemia. The five-second average around the nadir and peak values are defined as the minimal and maximal value, respectively. Reoxygenation amplitudes are calculated as the difference between the minimal and maximal value ($\Delta_{Recovery}$). O_2_Hb and dHb are measures of relative concentration change from zero, set at the start of the warm-up phase. As such, the absolute $\Delta_{Exercise}$ for O_2_Hb and dHb are equivalent to the minimal attained value. Figure 1 gives a visual explanation of these parameters.

#### 2.5.2. Kinetic Analysis

In this research, an empirical mono-exponential model was used to describe the reoxygenation kinetics during recovery from maximal exercise. This is a well-known approach that is frequently advised in the literature [31,32,33,34]. Figure 1 shows the original data together with the fitted curve. From the onset of the recovery, the following equation was used for fitting the data:

(2) $$Y\;\left(t\right)=Y_{Mininum}+Y\Delta_{Recovery}\times \left(1-e^{-\frac{t}{Tau}}\right)$$

where $Y_{\left(t\right)}$ is the exponential fit data from the NIRS-derived signal (either TSI, O_2_Hb, or dHb), $Y_{Minimum}$ is the minimum value, $Y\Delta_{Recovery}$ is the earlier described reoxygenation amplitude, and Tau is the empirical time constant related to the recovery. For curve fitting, a start- and endpoint are mandatory. In particular, in patients, the mono-exponential reoxygenation profile does not start immediately after exercise, instead it starts only after a certain time delay (Td). In order to avoid observer bias, we used an automated method to detect the start and end of the mono-exponential curve. Using a matched filter cross-correlation by sliding a pre-defined mono-exponential-shaped kernel, the start of the mono-exponential fit (and thus the end of the Td) was determined by the highest cross-correlation [32]. The endpoint is automatically detected as the peak value that is not exceeded by another value within 30 s. This time frame, including the 30 s following the peak value, was considered the time window for the fitting procedure. By fitting this model to the data using the least squares method, the model parameters were obtained. Finally, the sum of Td and Tau is the mean response time (MRT). Earlier, our group and others found that this MRT shows a significantly higher relative test–retest reliability than *Td* or *Tau*. This may be explained by physiological variability and/or the brief movement interruption from pedal detachment to the resting platform, resulting in ambiguous fitting parameters of *Td* and Tau around the inflection point of the mono-exponential function, while MRT includes both [19,35]. The goodness of mono-exponential fit was considered satisfactory when exceeding R^2^ > 0.85 (coefficient of determination) [36,37]. In addition to mono-exponential fitting, the half value time (HVT) was calculated, defined as the time for the NIRS signal to reach half the $\Delta_{Recovery}$ in the recovery period (Figure 1).

**Figure 2:**
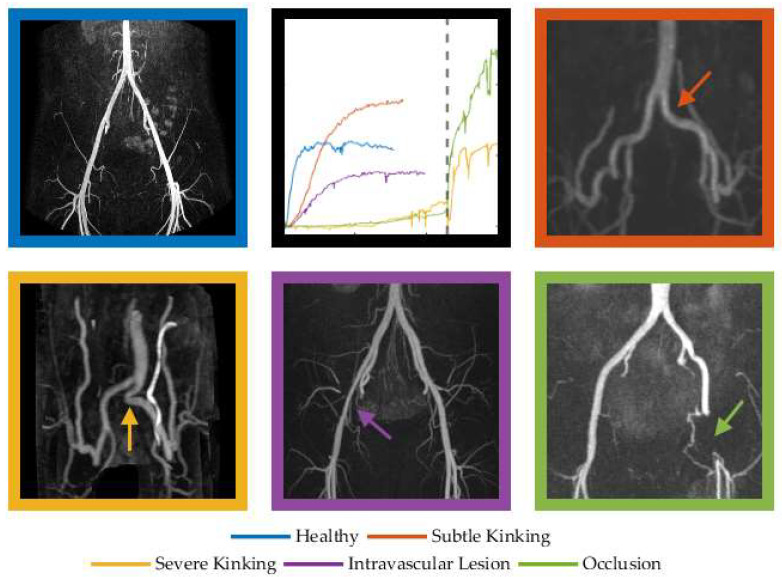
Reoxygenation patterns of O_2_Hb following maximal exercise in cases of graded- severity of FLIA. The dashed line represents a sudden change from competitive posture to normal posture to demonstrate the effect of severe kinking on oxygenation (orange) and immediate reoxygenation in the patient with the femoral occlusion (green). We postulate that this reoxygenation is caused by elevated hydrostatic pressure in the upright position and relief of the ‘functional occlusion’ of the collateral circulation in the flexed hip position.

In some instances, it was impossible to estimate the mono-exponential parameters due to a non-monoexponential recovery response or fit parameters lying outside the experimental time frame (e.g., unobservable MRT values in the analysis of 14–16 legs depending on the used NIRS signal (TSI, O_2_Hb, or dHb)). In such cases where a monoexponential function could not be fitted to the reoxygenation data, or where minimal reoxygenation was observed, it was thought that extreme functional kinking or arterial occlusion was restricting blood flow to almost zero (as collateral flow is minimal) while the competitive posture (including acute hip flexion) was maintained (Figure 2, purple line). We recently published case reports of such aberrant responses in a patient with femoral arterial occlusion and severe functional kinking [5,18]. Since excluding these cases would introduce bias, we included them and considered the non-observable HVT and/or MRT values.

### 2.6. Statistical Analysis

All statistical analyses were performed using R (version 4.2.1, R Foundation for Statistical Computing, Vienna, Austria) [38]. Normality of the data was assessed by visual evaluation of histograms and calculation of skewness and kurtosis values. In case of a normal distribution, characteristics and outcome parameters are presented as the mean value ± standard deviation (SD). If this assumption was violated, the median with interquartile range was reported (median (IQR_1_–IQR_3_)). To test significant differences between groups, the independent Student’s *t*-test was used. If the assumption of normality was violated, the Mann–Whitney *U*-test was used. Categorical data were analyzed using the chi-squared test.

#### Diagnostic Accuracy

To study if the newly introduced NIRS-derived kinetic variables did improve diagnostic accuracy compared to the use of ABI measures alone, we have fitted a logistic regression model. For all the three signals separately (TSI, O_2_Hb, and dHb), we have derived the corresponding kinetic variables and included them in the logistic model, together with possible confounders (i.e., age, sex, BMI, $W_{Peak/kg}$, ATT, $ABI_{Flexed}$). The variables eventually included in the model were selected by means of stepwise backward selection, leaving out the covariates with estimated coefficients with *p*-values larger than 0.10, starting from the ones with the highest *p*-value). The models were validated via a 10-fold cross-validation with stratification to balance the class distribution within the splits [39,40,41,42]. Since a number of kinetic and/or absolute values were censored (i.e., the recovery time was outside the window of observation, or no reoxygenation was seen at all, e.g., Figure 2 purple signal), we considered different types of analysis, including substitution method (SM) and a missing data indicator (MDI) approach. Since MRT and HVT are highly correlated within in each NIRS signal (TSIρ = 0.98, *p* < 0.001; O_2_Hb ρ = 0.97, *p* < 0.001; dHb ρ = 0.98, *p* < 0.001) and also between NIRS signals (e.g., TSI vs. O_2_Hb MRT ρ = 0.96, *p* < 0.001; HVT ρ = 0.97, *p* < 0.001), this would introduce multicollinearity in regression analysis, and thus they were included in two separate models [39,40,41,42]. Since one might be calculated while the other is censored (e.g., absence of mono-exponential reoxygenation), we provide the use of one or both models.

Firth’s bias reduction method was used to resolve the problem of separation in logistic regression (R package “brglm2”) [39,40,41,42]. A receiver operating characteristic (ROC) curve is used as a graphical approach to analyze the performance of the classifiers. Thereafter, three cut-off points are determined based on Youden index, the maximum potential effectiveness of a biomarker (sensitivity + specificity − 1); the maximal sensitivity around the 95% specificity value; and the maximal specificity around the 95% sensitivity value to provide multiple thresholds for multiple purposes (screening or diagnosis). From the estimates of the model parameters, the estimated probability of having FLIA can be calculated by the inverse logit function (1(1+e−x)). Since including both legs would lead to non-independent data, we choose to randomly select either the right or left leg of healthy subjects (*n* = 33) and patients with bilateral abnormalities and similar severity complaints for left and right (*n* = 15). The leg with the most severe complaints was chosen in patients with asymmetrical bilateral complaints (*n* = 91), and the affected leg was chosen in patients with unilateral complaints (*n* = 95). A *p*-value < 0.05 was considered statistically significant for all tests.

## 3. Results

A total of 33 healthy subjects and 201 patients fulfilled the study criteria. Healthy subjects were on average 14 years younger, attained a higher $W_{Peak/kg}$, suffered less often from back complaints, and were engaged in a lower level of competition (Table 1).

The modified ABI_Flexed_ was normal (>0.54) in both groups (healthy 0.74 (0.62–0.79) versus patients 0.58 (0.47–0.68)), although these data suggested a fair number of subtle flow limitations in these individuals. Indeed, the ABI_Flexed_ in patients was significantly lower compared to healthy controls (*p* < 0.001). Healthy subjects were able to attain a significantly higher maximal workload (5.9 (0.9) vs. 5.1 (1.1), *p* < 0.05).

Significantly higher values of PSV_Extended_, PSV_Flexed_, and PSV_Psoas_ (m/s) contraction were found in patients (Table 2).

### 3.1. Diagnostic Use of Near-Infrared Spectroscopy

#### 3.1.1. Statistical Considerations

The analyses of simple complete case logistic regression confirmed that the predictive performance of isolated absolute and signal amplitude variables were around the diagonal line of the ROC curve and revealed no significant improvement in AUC when combined with kinetics. In addition, we found no differences in AUC between SM or MDI methods [42,43]. In hindsight, the likelihood of finding a censored value in a healthy subject is practically zero, and, therefore, it is not useful to employ maximum likelihood estimations for censored variables. We have, therefore, chosen to substitute the non-observed values (greater than the experiment time of five minutes) with the maximum value itself (i.e., 300 s).

#### 3.1.2. Diagnostic Accuracy of NIRS

Neither adding absolute values nor amplitude values for NIRS showed significant differences between healthy controls and patients. In contrast, highly significant differences were found in all kinetic parameters (Table 3). The results of logistic regression after substituting the censored values showed that after backward stepwise regression, the model had the largest AUC in dHb for MRT (AUC 0.939; threshold 0.843) and HVT (AUC 0.937; threshold 0.839) with both a sensitivity and specificity of 0.80 and 0.93, respectively. Table S1 of the Supplementary Materials provides the details on the different models and the estimated coefficients.

#### 3.1.3. Comparing ABI_Flexed_ with NIRS

**Figure 3:**
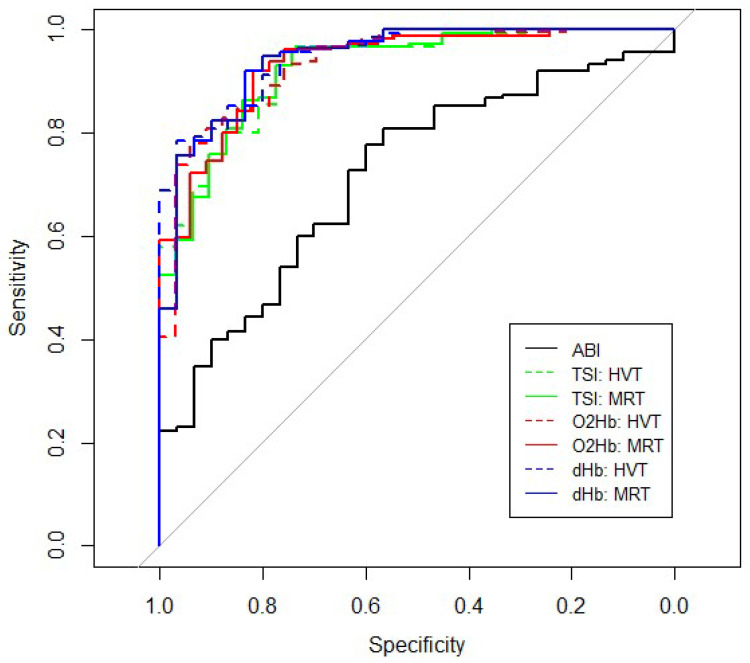
Receiver operating characteristic (ROC)-curve of ABI_Flexed_ and reoxygenation kinetics of the HVT and MRT for TSI, O_2_Hb, and dHb.

ABI_Flexed_ in healthy subjects and patients were mainly in the normal range. However, between group differences were significant (healthy: median 0.74 (IQR, 0.62–0.79) vs. 0.58 (0.47–0.68) *p* < 0.001). Compared with NIRS, ABI_Flexed_ values alone gave rather disappointing results with an AUC of only 0.717 with a threshold of 0.765, sensitivity of 0.78, and a specificity of 0.60 at the Youden Index (Figure 3; Table 4). However, combining ABI_Flexed_ with NIRS kinetic resulted in an improved model in both the TSI and O_2_Hb signal (AUC 0.921–0.934; sensitivity 0.80–0.96; specificity 0.74–0.91; all in ranges).

## 4. Discussion

The diagnosis of a sport-related flow limitation in the iliac artery (FLIA) is a major challenge. The diagnostic accuracy of current used tools are suboptimal, especially in the early stages where the changes in blood flow are subtle. In an earlier study, using values of ABI_Flexed_ after exercise alone, a quarter of the athletes with unilateral FLIA are misclassified. In bilateral FLIA, more than half of the patients do not breech the diagnostic threshold [2,3]. The present study investigated whether NIRS characteristics that were obtained during recovery after a maximal exercise test had additional diagnostic value when compared to and combined with ABI_Flexed_ values. Using the Youden index, the sensitivity of a range of NIRS characteristics varied from 0.80 to 0.96, whereas specificity was between 0.74 and 0.93. For screening purposes, we have set a high sensitivity and found that the use of the kinetic variable MRT of the dHb signal is advised with a 0.94 sensitivity and a 0.73 specificity. For confirming the diagnosis, either HVT or MRT of dHb with a specificity of 0.93 and a sensitivity of 0.80 is recommended.

The present study suggests that NIRS substantially augments the diagnostic accuracy of FLIA. However, this is not in line with research by Julliene et al. who found no significant difference in a variety of NIRS parameters, when comparing cyclists with symptomatic endofibrosis to healthy controls [44]. Differences in methods may explain this discrepancy. Their study did not look for asymptomatic vascular abnormalities in healthy subjects using echo-Doppler, possibly introducing bias. In addition, individuals were studied in a recumbent posture, whereas arterial kinking is more pronounced in a competitive cycling body position. Moreover, they did not use the mono-exponential fitting of reoxygenation kinetics, a technique that is thought to provide important diagnostic information [16,17]. Finally, they subtracted values in the symptomatic leg from those of the asymptomatic leg. Our results show that this approach might give substantial bias as pure unilateral cases are scarce and often bilateral involvement is underestimated using current diagnostic tools with relatively low sensitivity and specificity. For instance, our study found that seemingly non-affected legs of patients had significantly different reoxygenation kinetics from legs the of healthy subjects. These findings suggest that asymptomatic patient legs may not be entirely unaffected and that the current tools lack sensitivity in individuals having very subtle arterial abnormalities.

During this study, we found that NIRS kinetic parameters show significant differences between healthy subjects and our patient population, while absolute NIRS values and amplitudes did not in most cases. In patients with unilateral FLIA, significant differences were identified between affected and non-affected legs using paired measures testing, with affected legs reaching lower minimal TSI during exercise and higher maximal TSI, O_2_Hb, and dHb during the recovery phase (Table 3). These small absolute differences indicate that the tissue metabolic state between legs may be similar at maximal exercise tolerance and at peak hyperemia during recovery. Directionally, lower TSI at maximal exercise may be considered a proxy for greater O_2_ extraction [45,46], while higher peak O_2_Hb and dHb during recovery is consistent with a stronger vasodilatory stimulus [46,47]. This may point to the affected leg being more metabolically disrupted than the non-affected leg, which would be consistent with blood flow limitation. However, because absolute NIRS values and amplitudes were on the whole not different between patients and healthy subjects, we believe they are not useful in the diagnosis of FLIA.

During near-maximal exercise, O_2_ delivery is not able to meet energetic requirements, and so local O_2_ uptake is constrained by O_2_ delivery even in healthy legs. Although absolute NIRS values between patients and healthy subjects were not different on the whole, these values were reached at a significantly lower power output (W_Peak/kg_) in patients, despite patients having a longer training history and performing at a higher competitive level, suggesting higher fitness than healthy subjects (Table 1). We hypothesize that a lower peak power output could be explained by a greater relative O_2_ delivery limitation in patients, which would constrain total work output and lead to earlier fatigue and task failure. A similar rationale could explain close NIRS absolute values in unilateral patients at maximal exercise: the affected leg has produced less power, thus incurring a lower energetic demand for O_2_ than the non-affected leg. This is a common observation, as shown in our study, in unilateral FLIA patients, who made up the majority of patients in this study, when measuring left and right pedal power independently [18].

Once maximal exercise tolerance is reached, the energetic demands of workload are removed and both O_2_ delivery and uptake will begin to recover. During recovery, O_2_ delivery exceeds O_2_ uptake, and so a net positive reoxygenation is observed by NIRS in the local tissue (Figure 1). However, the delayed reoxygenation kinetics observed in the patient group (Figure 2 and Table 1) indicate that there is less excess O_2_ delivery during recovery, i.e., higher fractional O_2_ extraction and slower reconstitution of the local metabolic milieu. This is consistent with blood flow limitation at the conduit iliac artery. It is well established that NIRS reoxygenation is faster in fitter subjects [48,49] and delayed in clinical conditions, such as peripheral vascular disease [14,15,16,17,50]. The present study demonstrates that NIRS kinetic parameters may be valuable in the diagnosis of FLIA.

Several limitations should be acknowledged. First, a small number of female patients were included in this study. Historically, fewer women have trained and competed at the highest levels of strenuous exercise, which may have resulted in lower rates of vascular damage. Moreover, a fair number of female patients were excluded because of their skinfold thickness, which makes this method (and NIRS devices in general) unfortunately less applicable for female athletes. A correction method for ATT based on previous literature or using occlusion calibrations may allow for more accurate analysis of NIRS data collected in females and older individuals [11,51]. Secondly, our vascular technician was aware that the healthy subjects had no complaints, which might potentially have biased duplex Doppler echography interpretations. However, absolute measures, such as PSV and diameter measurements, were also used along with subjective interpretation for the presence of kinking and intravascular lesion. Therefore, concerning the vascular tree, only true healthy subjects were included and doubtful cases were excluded. Concerning patients, all had typical complaints and abnormalities on echo-Doppler. Third, our patient group is significantly older than the control group, although this was corrected for in the predictive model. It has been shown that after maximal exercise, NIRS reoxygenation is more strongly associated with fitness level than with age per se [49]. Our patient group was older but also performed at a higher competitive level and had a longer training history on average (Table 1), indicating that they may have been at higher fitness and training status than our healthy subject group. Fourth, in the early phases of this research, we used a maximum of five minutes recovery instead of a clear reoxygenation maximum followed by a distinct decrease in the oxygenation. This resulted in recordings that were involuntarily ended before a stable end-point. Therefore, reoxygenation kinetics could not be calculated (*n* = 6 to *n* = 22, depending on the signal used for analysis). Finally, there were hardware limitations in data collection related to loss of optical signal in highly vascularized tissues related to the extreme physical fitness of our patient population. This phenomenon results in (near) complete absorption of the optical spectra, and, as a consequence, insufficient light intensity by the photodetector. The greatest source-detector distance often did not produce a reliable signal, which affected the interpretation across the three combined signals. As such, the first two LED pairs (30–35 mm source-detector distance) were used for all datasets, as per recommendation of the manufacturer. Some data still required exclusion from analysis (TSI 11.6–14.7%; O_2_Hb 0.0%, and dHb 11.3–15.2%). The NIRS manufacturer expects that this drawback will be mitigated with a new feature called multi power gain control, which automatically enhances the exposure time of these LEDs, if needed. Other technical limitations are extensively described in our research assessing test–retest reliability [19].

Future research should investigate NIRS reoxygenation kinetics in FLIA patients with isolated arterial kinking and in patients with endofibrosis to evaluate whether greater delays can be related to a more advanced progression of FLIA. Additionally, it is consistently observed that maximal performance is impaired in athletes with FLIA when performing an incremental ramp test lasting 8–12 min in the laboratory. However, affected athletes often report impairment at lower workloads in real-world training and competition over longer durations or when performing intermittent efforts. Future research can use muscle oxygenation in combination with systemic physiological measurements (ventilation, pulmonary gas exchange, heart rate) to investigate impairment as a function of both intensity and duration, which may be useful in athlete monitoring. Finally, although NIRS has already proved it has a very good diagnostic value future, research into the combination with pedal power measurements may even lead to further improvement of the diagnostic value of exercise testing [21].

## 5. Conclusions

This is the first study examining the diagnostic accuracy of NIRS using reoxygenation kinetics in a large group of individuals with unilateral and bilateral sport-related iliac artery flow limitations. Our study indicates that delayed reoxygenation kinetics is an indicator of an underlying sport-related flow limitation. NIRS-derived kinetic parameters combined with ABI_Flexed_ substantially augment diagnostic accuracy above ABI_Flexed_ alone.

## Figures and Tables

**Table 1:** Characteristics of healthy subjects and athletes suffering from FLIA (data are presented as mean (SD) or median (IQR_1_–IQR_3_)).

	Healthy Subjects (*n* = 33)	Patients (*n* = 201)
Age (years)	26 (21–38)	40 (28–51) *
Sex		
Male	30 (90.9%)	177 (88.1%)
Female	3 (9.1%)	24 (11.9%)
Cardiovascular family history		
Yes	6 (18.2%)	33 (16.4%) †
No	27 (81.8%)	167 (83.1%)
Smoking (history)		
Yes	0 (0.0%)	3 (1.5%)
No	33 (100.0%)	198 (98.5%)
Ex-smoker	2 (6.1%)	23 (11.4%)
Length (cm)	181.1 (8.2)	180.9 (8.1)
Weight (kg)	73 (67–77)	73 (68–82)
BMI (kg/m^2^)	21.9 (20.6–23.1)	22.3 (20.9–24.2)
Cycled lifetime (km)	140,000 (69,000–263,000)	200,000 (110,000–350,000)
Years of cycling (years)	11 (6–15)	15 (10–26) *
Level		
Professional	7 (21.2%)	56 (27.9%) **
Competitive	18 (54.5%)	50 (24.9%)
Recreational	8 (24.2%)	95 (47.3%)
Type of sport(s) (multiple answers possible)		
Cycling	33 (100.0%)	186 (92.5%)
Ice speed skating	1 (3.0%)	22 (10.9%)
Triathlon	3 (9.1%)	13 (6.5%)
Running	3 (9.1%)	26 (12.9%)
Other	0 (0.0%)	4 (2.0%)
Back-complaints		
Yes	8 (24.2%)	122 (60.7%) *
No	25 (75.8%)	79 (39.3%)
If yes, in relation to FLIA ‡	No FLIA	53 (43.44%)
FLIA side		
Left	No FLIA	76 (37.8%)
Right		19 (9.5%)
Both		106 (52.7%)

* *p* < 0.05; ** *p* < 0.05, post hoc analysis revealed asymmetrical differences in the competitive and recreational group; † One patient had no information of family history for cardiovascular disease; ‡ Counts with proportion for those who had back complaints.

**Table 2:** Doppler echography of iliac arteries in healthy subjects and FLIA patients.

	Healthy Subjects (*n* = 33)	Patients (*n* = 201)	
		Non-Affected Legs	Affected Legs
PSV_Extended_ (m/s)	0.78 (0.70–0.88)	1.12 (0.91–1.34) *	1.40 (1.03–1.79) †‡
PSV_Flexed_ (m/s)	0.95 (0.80–1.06)	1.40 (1.08–1.74) *	2.02 (1.65–2.48) †‡
PSV_Psoas_ (m/s)	1.02 (0.87–1.19)	1.54 (1.15–1.72) *	2.21 (1.81–2.67) †‡
Presence of arterial kinking			
Yes	No FLIA		184 (91.5%)
No			17 (8.5%)
Location of arterial kinking			
CIA	No FLIA		105 (52.2%)
EIA			130 (64.7%)
IIA			1 (0.5%)
CFA			0 (0.0%)
Presence of an intravascular lesion			
Yes	No FLIA		118 (58.7%)
No			83 (41.3%)
Location of the intravascular lesion			
CIA	No FLIA		37 (18.4%)
EIA			87 (43.3%)
IIA			0 (0.0%)
CFA			5 (2.5%)

Normal values for PSV_Extended_ < 1.48 m∙s^−1^, for PSV_Flexed_ and PSV_Psoas_ < 1.70 m∙s^−1^ [2,3,20]. * Significant difference between healthy subjects and the patients’ non-affected leg *p* < 0.05. † Significant difference between healthy subjects and the patients’ affected leg *p* < 0.05. ‡ Significant difference between the patients’ non-affected leg and the patients’ affected leg *p* < 0.05. CIA—common iliac artery; EIA—external iliac artery; IIA—internal iliac artery; CFA—common femoral artery.

**Table 3:** Characteristics of NIRS in healthy subjects and FLIA patients. The non-affected legs of patients with unilateral FLIA are reported as informative but not included in the diagnostic model.

		Healthy Subjects		Patients				
				Unilateral (Complete Cases)			All Included Affected Patient Legs	
		*n*_Healthy_	Healthy Legs	*n*_Unilateral_	Non-Affected Leg	Affected Leg	*n*_Affected_	Affected Leg
*Cycling test*								
	W_Peak/kg_	33	5.9 (0.9)	95	5.2 (1.0) *		201	5.1 (1.1) *
	ABI_Flexed_	33	0.74 (0.62–0.79)	94	0.67 (0.59–0.74)	0.58 (0.44–0.68) *†	200	0.58 (0.47–0.68) *†
*Absolute values and amplitudes*								
TSI	Baseline (%)	32	60.6 (58.1–64.0)	87	59.5 (53.8–63.8)	59.1 (54.6–63.4)	188	58.5 (54.5–62.7) *
	Minimal (%)	30	43.2 (39.0–46.0)	71	43.6 (38.6–47.4)	43.0 (33.4–46.6) †	166	40.5 (34.3–45.9) †
	Maximal (%)	33	67.7 (3.9)	72	66.9 (4.4)	67.9 (4.7) †	169	67.6 (4.7)
	$\Delta_{Exercise}\;\left(\%\right)$	31	18.1 (14.1–23.4)	73	16.6 (11.6–21.2)	18.2 (13.5–23.1) †	167	17.5 (13.5–23.3) †
	$\Delta_{Recovery}\;\left(\%\right)$	31	25.6 (21.1–29.8)	54	23.8 (18.8–28.7)	24.9 (18.9–33.7) †	141	25.4 (20.9–33.9)
O_2_Hb	Maximal (a.u.)	33	7.9 (2.4–12.5)	77	7.2 (3.0–10.6)	9.1 (5.5–14.7) †	170	9.0 (4.5–13.5)
	$\Delta_{Exercise}\;\left(a.u.\right)$ ‡	33	14.8 (12.8–18.8)	92	14.1 (10.8–20.3)	14.8 (11.2–20.3)	199	15.1 (11.2–19.9)
	$\Delta_{Recovery\;}\left(a.u.\right)$	33	25.4 (17.8–30.1)	77	21.4 (16.7–28.2)	25.6 (15.9–34.8) †	170	23.9 (16.7–33.0)
dHb	Maximal (a.u.)	32	9.2 (8.6)	69	8.2 (8.2)	11.2 (9.2) †	157	10.3 (8.6)
	$\Delta_{Exercise\;}\left(a.u.\right)$ ‡	30	25.1 (20.1–32.6)	70	24.6 (19.3–29.1)	24.3 (19.6–32.5)	165	24 (17.7–31.6)
	$\Delta_{Recovery}\;\left(a.u.\right)$	30	37.2 (23.8–45.9)	54	30.4 (24.4–38.2)	33.8 (23.2–43.7) †	133	32 (22.7–41.4)
*Kinetic variables*								
TSI	Tau (s)	33	15.2 (10.9–21.0)	59	21.0 (17.5–32.6) *	34.0 (20.2–48.7) *†	146	33.5 (20.3–50.2) *†
	Td (s)	33	9.8 (1.9–15.5)	59	17.8 (12.1–26.5) *	23.4 (14.4–35.5) *†	146	23.1 (15.1–36.3) *†
	MRT (s)	33	22.9 (17.5–31.4)	59	37.4 (29.2–60.4) *	58.2 (39.1–83.5) *†	146	57.5 (38.6–86.5) *†
	HVT (s)	31	17.8 (15.6–27.4)	54	30.3 (25.0–50.2) *	45.4 (28.7–61.6) *†	142	45.4 (30.5–65.2) *†
O_2_Hb	Tau (s)	33	20.9 (16.8–26.9)	81	30.9 (21.0–43.0) *	38.5 (28.2–62.3) *†	180	39.6 (28.0–59.9) *†
	Td (s)	33	8.6 (2.9–15.5)	81	17.0 (11.6–22.8) *	21.7 (15.6–34.1) *†	180	21.6 (15.6–33.7) *†
	MRT (s)	33	29.1 (22.2–38.7)	81	45.9 (36.3–65.0) *	61.6 (45.5–100.0) *†	180	62.0 (45.8–93.7) *†
	HVT (s)	33	23.9 (19.4–29.9)	77	35.0 (27.8–50.2) *	47.5 (34.5–72.0) *†	169	47.7 (35.9–71.4) *†
dHb	Tau (s)	33	18.7 (15.5–28.2)	57	31.0 (22.3–44.8) *	41.2 (27.0–59.5) *†	142	43.0 (27.9–62.7) *†
	Td (s)	33	11.1 (4.9–16.4)	57	18.8 (12.5–25.1) *	24.7 (16.8–35.5) *†	142	23.0 (15.7–35.3) *†
	MRT (s)	33	28.7 (22.4–40.3)	57	47.2 (38.3–68.2) *	64.9 (45.8–92.4) *†	142	65.2 (47.7–98.9) *†
	HVT (s)	30	22.6 (19.8–29.5)	54	35.6 (29.1–59.6) *	50.4 (33.9–72.7) *†	133	50.0 (36.2–76.9) *†

* Significantly different from healthy subjects *p* < 0.05. † Significantly different from unilateral non-affected legs *p* < 0.05. ‡ $\Delta_{Exercise}\;\left(a.u.\right)$ is the absolute equivalent of the minimal attained value while O_2_Hb and dHb are set to zero at the start of the exercise test.

**Table 4:** ROC characteristics with the thresholds, sensitivities and specificities.

		AUC	T ^1^	Sens ^1^	Spec ^1^	T ^2^	Sens ^2^	Spec ^2^	T ^3^	Sens ^3^	Spec ^3^
ABI		0.717 (0.626–0.807)	0.765	0.78	0.60	0.659	0.94	0.10	0.900	0.23	0.97
TSI	HVT	0.924 (0.885–0.963)	0.570	0.96	0.74	0.618	0.95	0.74	0.933	0.65	0.97
	MRT	0.921 (0.881–0.961)	0.603	0.95	0.74	0.573	0.95	0.65	0.919	0.68	0.97
O_2_Hb	HVT	0.934 (0.901–0.968)	0.646	0.94	0.82	0.613	0.96	0.70	0.962	0.63	0.97
	MRT	0.932 (0.898–0.966)	0.864	0.80	0.91	0.623	0.96	0.70	0.917	0.72	0.97
dHb	HVT	0.937 (0.902–0.973)	0.839	0.80	0.93	0.619	0.94	0.73	0.963	0.59	0.97
	MRT	0.939 (0.904–0.974)	0.843	0.80	0.93	0.538	0.95	0.70	0.875	0.75	0.97

^1^ Based on the Youden Index. ^2^ Highest specificity with a minimal sensitivity of 0.95. ^3^ Highest sensitivity with a minimal sensitivity of 0.95.

## Data Availability

The data that support the findings of this study are available from the corresponding author upon reasonable request.

## Supplementary Materials

The following supporting information can be downloaded at: https://www.mdpi.com/article/10.3390/jcm11247462/s1, Table S1: Information on remaining estimates for the different derived NIRS signals (TSI, O2Hb and dHb) and kinetic variables (HVT and MRT) after backward elimination.

## References

[1] Bender M.H., Schep G., de Vries W.R., Hoogeveen A.R., Wijn P.F. (2004). Sports-related flow limitations in the iliac arteries in endurance athletes: Aetiology, diagnosis, treatment and future developments. Sport. Med..

[2] Schep G., Bender M.H., Schmikli S.L., Mosterd W.L., Hammacher E.R., Scheltinga M., Wijn P.F. (2002). Recognising vascular causes of leg complaints in endurance athletes. Part 2: The value of patient history, physical examination, cycling exercise test and echo-Doppler examination. Int. J. Sport. Med..

[3] Schep G., Schmikli S.L., Bender M.H., Mosterd W.L., Hammacher E.R., Wijn P.F. (2002). Recognising vascular causes of leg complaints in endurance athletes. Part 1: Validation of a decision algorithm. Int. J. Sport. Med..

[4] Abraham P., Bickert S., Vielle B., Chevalier J.M., Saumet J.L. (2001). Pressure measurements at rest and after heavy exercise to detect moderate arterial lesions in athletes. J. Vasc. Surg..

[5] Van Hooff M., Schep G., Bender M., Scheltinga M., Savelberg H. (2021). Sport-related femoral artery occlusion detected by near-infrared spectroscopy and pedal power measurements: A case report. Physician Sportsmed..

[6] Korsten-Reck U., Rocker K., Schmidt-Trucksass A., Schumacher Y.O., Striegel H., Rimpler H., Dickhuth H.H. (2007). External iliac artery occlusion in a young female cyclist. J. Sport. Med. Phys. Fit..

[7] Schep G., Bender M.H., van de Tempel G., Wijn P.F., de Vries W.R., Eikelboom B.C. (2002). Detection and treatment of claudication due to functional iliac obstruction in top endurance athletes: A prospective study. Lancet.

[8] Bender M.H., Schep G., Bouts S.W., Backx F.J., Moll F.L. (2012). Endurance athletes with intermittent claudication caused by iliac artery stenosis treated by endarterectomy with vein patch–short- and mid-term results. Eur. J. Vasc. Endovasc. Surg..

[9] Peach G., Schep G., Palfreeman R., Beard J.D., Thompson M.M., Hinchliffe R.J. (2012). Endofibrosis and kinking of the iliac arteries in athletes: A systematic review. Eur. J. Vasc. Endovasc. Surg..

[10] Abraham P., Chevalier J.M., Leftheriotis G., Saumet J.L. (1997). Lower extremity arterial disease in sports. Am. J. Sport. Med..

[11] Barstow T.J. (2019). Understanding near infrared spectroscopy and its application to skeletal muscle research. J. Appl. Physiol..

[12] Van Beekvelt M.C., Colier W.N., Wevers R.A., Van Engelen B.G. (2001). Performance of near-infrared spectroscopy in measuring local O_2_ consumption and blood flow in skeletal muscle. J. Appl. Physiol..

[13] Ferrari M., Muthalib M., Quaresima V. (2011). The use of near-infrared spectroscopy in understanding skeletal muscle physiology: Recent developments. Philos. Trans. A Math. Phys. Eng. Sci..

[14] Vardi M., Nini A. (2008). Near-infrared spectroscopy for evaluation of peripheral vascular disease. A systematic review of literature. Eur. J. Vasc. Endovasc. Surg..

[15] Boezeman R.P., Moll F.L., Unlu C., de Vries J.P. (2016). Systematic review of clinical applications of monitoring muscle tissue oxygenation with near-infrared spectroscopy in vascular disease. Microvasc. Res..

[16] Baltrunas T., Mosenko V., Mackevicius A., Dambrauskas V., Asakiene I., Rucinskas K., Narmontas P. (2022). The use of near-infrared spectroscopy in the diagnosis of peripheral artery disease: A systematic review. Vascular.

[17] Cornelis N., Chatzinikolaou P., Buys R., Fourneau I., Claes J., Cornelissen V. (2021). The Use of Near Infrared Spectroscopy to Evaluate the Effect of Exercise on Peripheral Muscle Oxygenation in Patients with Lower Extremity Artery Disease: A Systematic Review. Eur. J. Vasc. Endovasc. Surg..

[18] van Hooff M., Schep G., Meijer E., Bender M., Savelberg H. (2018). Near-Infrared Spectroscopy Is Promising to Detect Iliac Artery Flow Limitations in Athletes: A Pilot Study. J. Sport. Med..

[19] Van Hooff M., Meijer E.J., Scheltinga M.R.M., Savelberg H., Schep G. (2022). Test-retest reliability of skeletal muscle oxygenation measurement using near-infrared spectroscopy during exercise in patients with sport-related iliac artery flow limitation. Clin. Physiol. Funct. Imaging.

[20] Schep G., Bender M.H., Schmikli S.L., Wijn P.F. (2001). Color Doppler used to detect kinking and intravascular lesions in the iliac arteries in endurance athletes with claudication. Eur. J. Ultrasound.

[21] Kleinloog J.P.D., van Hooff M., Savelberg H., Meijer E.J., Schep G. (2019). Pedal power measurement as a diagnostic tool for functional vascular problems. Clin. Biomech..

[22] Buchheit M., Hader k., Alberto M.-V. (2012). Tolerance to high-intensity intermittent running exercise: Do oxygen uptake kinetics really matter?. Front. Physiol..

[23] Niemeijer V.M., Jansen J.P., van Dijk T., Spee R.F., Meijer E.J., Kemps H.M., Wijn P.F. (2017). The influence of adipose tissue on spatially resolved near-infrared spectroscopy derived skeletal muscle oxygenation: The extent of the problem. Physiol. Meas..

[24] Rickham P.P. (1964). Human Experimentation. Code of Ethics of the World Medical Association. Declaration of Helsinki. Br. Med. J..

[25] Fletcher G.F., Ades P.A., Kligfield P., Arena R., Balady G.J., Bittner V.A., Coke L.A., Fleg J.L., Forman D.E., Gerber T.C. (2013). Exercise standards for testing and training: A scientific statement from the American Heart Association. Circulation.

[26] O’Connor F.G. (2012). ACSM’s Sports Medicine: A Comprehensive Review.

[27] Gornik H.L., Garcia B., Wolski K., Jones D.C., Macdonald K.A., Fronek A. (2008). Validation of a method for determination of the ankle-brachial index in the seated position. J. Vasc. Surg..

[28] Patterson M.S., Chance B., Wilson B.C. (1989). Time resolved reflectance and transmittance for the non-invasive measurement of tissue optical properties. Appl. Opt..

[29] Grassi B., Quaresima V., Marconi C., Ferrari M., Cerretelli P. (1999). Blood lactate accumulation and muscle deoxygenation during incremental exercise. J. Appl. Physiol..

[30] Pearson R.K. (2002). Outliers in process modeling and identification. IEEE Trans. Control Syst. Technol..

[31] Jones A.M., Poole D.C. (2005). Oxygen Uptake Kinetics in Sport, Exercise and Medicine.

[32] Niemeijer V.M., Spee R.F., Jansen J.P., Buskermolen A.B., van Dijk T., Wijn P.F., Kemps H.M. (2017). Test-retest reliability of skeletal muscle oxygenation measurements during submaximal cycling exercise in patients with chronic heart failure. Clin. Physiol. Funct. Imaging.

[33] DeLorey D.S., Kowalchuk J.M., Paterson D.H. (2003). Relationship between pulmonary O_2_ uptake kinetics and muscle deoxygenation during moderate-intensity exercise. J. Appl. Physiol..

[34] DeLorey D.S., Kowalchuk J.M., Paterson D.H. (2004). Effect of age on O_2_ uptake kinetics and the adaptation of muscle deoxygenation at the onset of moderate-intensity cycling exercise. J. Appl. Physiol..

[35] Leclair E., Thevenet D., Reguem S.C., Borel B., Baquet G., Berthoin S., Mucci P. (2010). Reproducibility of Measurement of Muscle Deoxygenation in Children During Exercise. Pediatr. Exerc. Sci..

[36] De Groote P., Millaire A., Decoulx E., Nugue O., Guimier P., Ducloux G. (1996). Kinetics of oxygen consumption during and after exercise in patients with dilated cardiomyopathy. New markers of exercise intolerance with clinical implications. J. Am. Coll. Cardiol..

[37] Kemps H.M., De Vries W.R., Hoogeveen A.R., Zonderland M.L., Thijssen E.J., Schep G. (2007). Reproducibility of onset and recovery oxygen uptake kinetics in moderately impaired patients with chronic heart failure. Eur. J. Appl. Physiol..

[38] The R Foundation (2022). A Language and Environment for Statistical Computing, 4.2.1..

[39] Kosmidis I. (2021). brglm2: Bias Reduction in Generalized Linear Models. R Package Version 0.6, 2.

[40] Kosmidis I., Firth D. (2020). Jeffreys-prior penalty, finiteness and shrinkage in binomial-response generalized linear models. Biometrika.

[41] Kosmidis I., Kenne Pagui E.C., Sartori N. (2020). Mean and median bias reduction in generalized linear models. Stat. Comput..

[42] Chiou S.H., Betensky R.A., Balasubramanian R. (2019). The missing indicator approach for censored covariates subject to limit of detection in logistic regression models. Ann. Epidemiol..

[43] Turkson A.J., Ayiah-Mensah F., Nimoh V. (2021). Handling Censoring and Censored Data in Survival Analysis: A Standalone Systematic Literature Review. Int. J. Math. Math. Sci..

[44] Julienne T., Ammi M., Hersant J., Henni S., Abraham P. (2018). Near-infrared spectroscopy of the thigh fails to discriminate cyclists with arterial endofibrosis from normal asymptomatic athletes. Vasc. Investig. Ther..

[45] Bruno G., Valentina Q. (2016). Near-infrared spectroscopy and skeletal muscle oxidative function *in vivo* in health and disease: A review from an exercise physiology perspective. J. Biomed. Opt..

[46] Martin-Rincon M., Gelabert-Rebato M., Perez-Valera M., Galvan-Alvarez V., Morales-Alamo D., Dorado C., Boushel R., Hallen J., Calbet J.A.L. (2021). Functional reserve and sex differences dursing exercise to exhaustion revealed by post-exercise ischaemia and repeated supramaximal exercise. J. Physiol..

[47] Stöcker F., Von Oldershausen C., Paternoster F.K., Schulz T., Oberhoffer R. (2016). Relationship of post-exercise muscle oxygenation and duration of cycling exercise. BMC Sport. Sci. Med. Rehabil..

[48] Ichimura S., Murase N., Osada T., Kime R., Homma T., Ueda C., Nagasawa T., Motobe M., Hamaoka T., Katsumura T. (2006). Age and Activity Status Affect Muscle Reoxygenation Time after Maximal Cycling Exercise. Med. Sci. Sport. Exerc..

[49] Meyer P.H., Missao E.T., McCully K. (2021). Muscle Oxidative Capacity in the Arms and Legs of Various Types of Endurance Trained Athletes. Med. Res. Arch..

[50] Stavres J., Sica C.T., Blaha C., Herr M., Wang J., Pai S., Cauffman A., Vesek J., Yang Q.X., Sinoway L.I. (2019). The exercise pressor reflex and active O_2_ transport in peripheral arterial disease. Physiol. Rep..

[51] Craig J.C., Broxterman R.M., Wilcox S.L., Chen C., Barstow T.J. (2017). Effect of adipose tissue thickness, muscle site, and sex on near-infrared spectroscopy derived total-[hemoglobin + myoglobin]. J. Appl. Physiol..

