# Supplementary material for: Diagnosing Sport-Related Flow Limitations in the Iliac Arteries Using Near-Infrared Spectroscopy
Source: J Clin Med. 2022 Dec 16;11(24):7462. doi: 10.3390/jcm11247462 (PMC9786904; doi:10.3390/jcm11247462)
Supplement: Supplementary file 1 [file jcm-11-07462-s001.zip › jcm-2074434-supplementary.pdf]

Supplementary Table S1. Information on remaining estimates for the different derived NIRS signals (TSI, [O_2Hb] and [dHb]) and kinetic variables (HVT and MRT) after backward elimination.

		Coefficients	Estimate	Std. Error	p-value
TSI	HVT	(Intercept)	9.53461	3.17146	0.003
		Sex_{Female}	2.39146	1.13186	0.035
		ABI_{Flexed}	-5.12423	2.43391	0.035
		$W_{Peak/kg}$	-1.43729	0.34154	<0.001
		HVT	0.09636	0.02756	<0.001
		Sex_{Female}*HVT	-0.09767	0.02800	<0.001
	MRT	(Intercept)	9.90632	3.23715	0.002
		Sex_{Female}	2.20779	1.13438	0.052
		ABI_{Flexed}	-5.29644	2.51439	0.035
		$W_{Peak/kg}$	-1.43495	0.34029	<0.001
		MRT	0.06894	0.02065	<0.001
		Sex_{Female}*MRT	-0.07116	0.02127	<0.001
[O_2Hb]	HVT	(Intercept)	-3.07754	1.97631	0.119
		Sex_{Female}	4.67446	1.35378	<0.001
		Age	0.10166	0.02406	<0.001
		ABI_{Flexed}	-5.75989	2.51581	0.022
		HVT	0.13284	0.03171	<0.001
		Sex_{Female}*HVT	-0.13104	0.03240	<0.001
	MRT	(Intercept)	-4.31993	2.19722	0.049
		Sex_{Female}	5.34278	1.52068	<0.001
		Age	0.10748	0.02528	<0.001
		ABI_{Flexed}	-5.19851	2.54351	0.041
		MRT	0.12004	0.02890	<0.001
		Sex_{Female}*MRT	-0.11795	0.02957	<0.001
[dHb]	HVT	(Intercept)	-7.52343	1.70135	<0.001
		Sex_{Female}	5.89421	1.54868	<0.001
		Age	0.08021	0.02216	<0.001
		MRT	0.17300	0.04019	<0.001
		Sex_{Female}*MRT	-0.16481	0.04105	<0.001
	MRT	(Intercept)	-8.38666	1.96237	<0.001
		Sex_{Female}	6.55573	1.76820	<0.001
		Age	0.08302	0.02330	<0.001
		MRT	0.15549	0.03723	<0.001
		Sex_{Female}*MRT	-0.14723	0.03811	<0.001
